# Betulinic acid self-assembled nanoparticles for effective treatment of glioblastoma

**DOI:** 10.1186/s12951-022-01238-7

**Published:** 2022-01-21

**Authors:** Yong Li, Yixuan Wang, Lun Gao, Yinqiu Tan, Jiayang Cai, Zhang Ye, Ann T. Chen, Yang Xu, Linyao Zhao, Shiao Tong, Qian Sun, Baohui Liu, Shenqi Zhang, Daofeng Tian, Gang Deng, Jiangbing Zhou, Qianxue Chen

**Affiliations:** 1 Department of Neurosurgery, Renmin Hospital of Wuhan University, Wuhan, Hubei 430060 People’s Republic of China; 2 Department of Neurosurgery, Yale University, New Haven, CT 06510 USA

**Keywords:** Betulinic acid, Nanoparticles, Proliferation, Apoptosis, CB1/CB2

## Abstract

**Background:**

Glioblastoma (GBM) is the most common and fatal primary tumor in the central nervous system (CNS). Due to the existence of blood–brain barrier (BBB), most therapeutics cannot efficiently reach tumors in the brain, and as a result, they are unable to be used for effective GBM treatment. Accumulating evidence shows that delivery of therapeutics in form of nanoparticles (NPs) may allow crossing the BBB for effective GBM treatment.

**Methods:**

Betulinic acid NPs (BA NPs) were synthesized by the standard emulsion approach and characterized by electron microscopy and dynamic light scattering analysis. The resulting NPs were characterized for their anti-tumor effects by cell viability assay, EdU-DNA synthesis assay, cell cycle assay, mitochondrial membrane potential, and PI-FITC apoptosis assay. Further mechanistic studies were carried out through Western Blot and immunostaining analyses. Finally, we evaluated BA NPs in vivo for their pharmacokinetics and antitumor effects in intracranial xenograft GBM mouse models.

**Results:**

BA NPs were successfully prepared and formed into rod shape. BA NPs could significantly suppress glioma cell proliferation, induce apoptosis, and arrest the cell cycle in the G0/G1 phase in vitro. Furthermore, BA NPs downregulated the Akt/NFκB-p65 signaling pathway in a concentration dependent manner. We found that the observed anti-tumor effect of BA NPs was dependent on the function of CB1/CB2 receptors. Moreover, in the intracranial GBM xenograft mouse models, BA NPs could effectively cross the BBB and greatly prolong the survival time of the mice.

**Conclusions:**

We successfully synthesized BA NPs, which could cross the BBB and demonstrated a strong anti-tumor effect. Therefore, BA NPs may potentially be used for effective treatment of GBM.

**Graphical Abstract:**

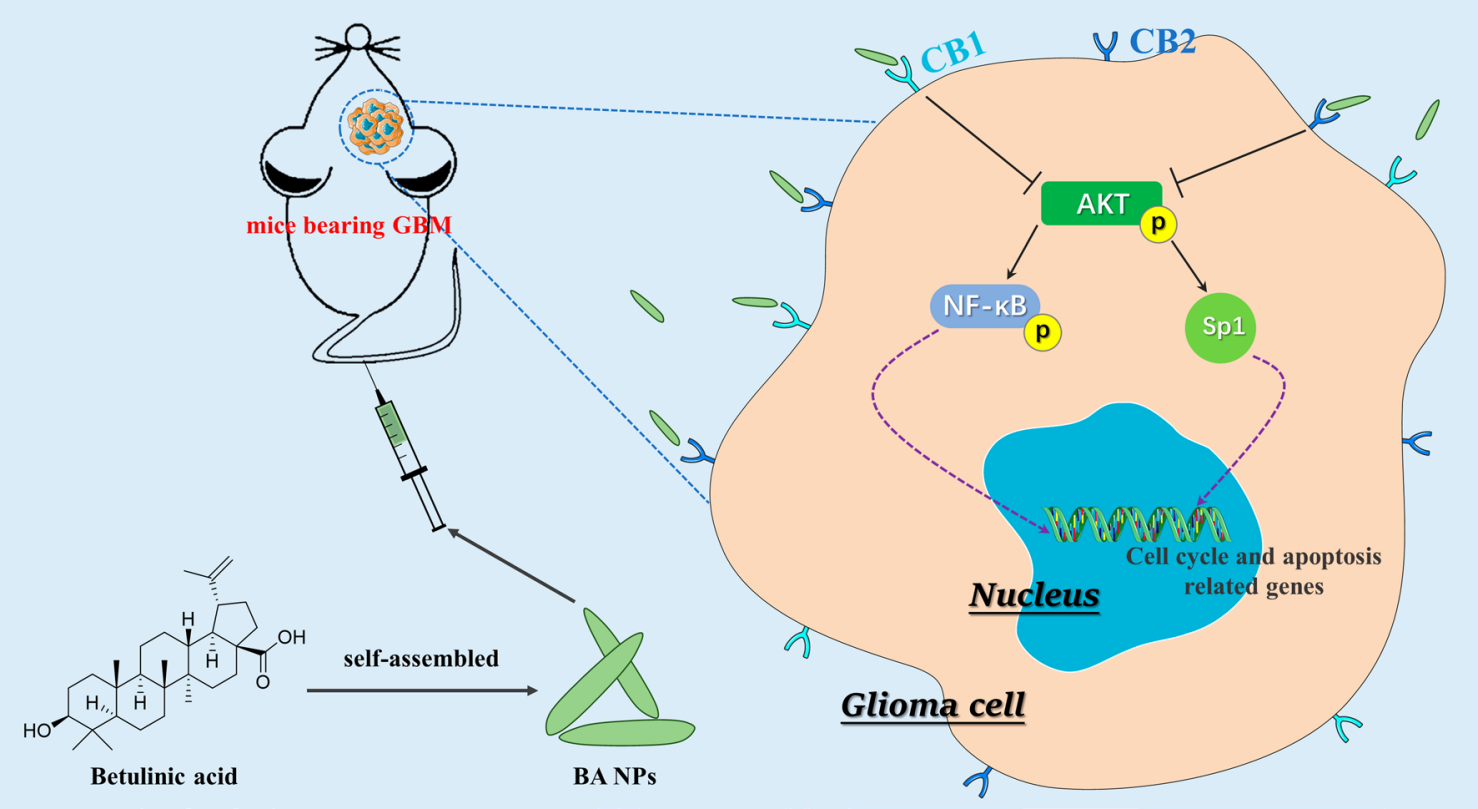

**Supplementary Information:**

The online version contains supplementary material available at 10.1186/s12951-022-01238-7.

## Introduction

Glioblastoma (GBM) is the most common, malignant, and fatal primary tumor in the central nervous system (CNS), accounting for approximately 50% of all gliomas [1]. Currently, the standard treatment for GBM is surgical resection, combined with postoperative radiotherapy and chemotherapy, to eliminate the tumor and delay its recurrence [2]. However, due to the blood-brain barrier (BBB), almost all macromolecular drugs and 98% of small molecule drugs cannot enter the brain, which makes it difficult for conventional oral or intravenous drugs to reach sufficient concentrations in the brain parenchyma [3–5]. Increasing the dose of glioma-treating drugs has been shown to result in increased systemic toxicity and is not an option for GBM treatment [6]. Currently, temozolomide (TMZ) is the main chemotherapy drug for GBM. However, at least 50% of GBM patients do not respond to TMZ or develop resistance after treatment with TMZ, which limits its overall effectiveness [7]. Therefore, identifying a drug that can efficiently pass through the systemic circulation, cross the BBB, and enter into the tumor parenchyma is urgently needed for improved GBM treatment.

Betulinic acid (BA) is a natural pentane pentacyclic triterpenoid that has been shown to have various biological functions, such as functioning as antioxidants, or having anti-inflammatory and anti-tumor effects [8–12]. It was shown that BA has a strong selective cytotoxicity to melanoma cells. Subsequently, it was found that BA has a wide range of anti-cancer activity against neuroblastoma, ovarian cancer, cervical cancer, leukemia and other tumor cells [13]. Currently, it has been determined that the anti-cancer mechanism of BA is to induce the reduction of mitochondrial membrane potential and the release of mitochondrial cytochrome C and Smac genes, which results in the activation of caspase-3 and induction of tumor cell apoptosis [14]. Recently, BA has been hypothesized to be a promising drug for the treatment of diseases in the brain, due to its specificity in killing GBM cells, but not the normal brain tissue [15, 16]. However, because of its poor water solubility, short half-life in vivo, poor tissue targeting, and inability to cross the BBB, its therapeutic potential cannot be capitalized [17].

We recently developed a novel approach to form nanoparticles using various naturally existing compounds, including BA [18–20]. In a previous study, we found that BA was able to self-assembled into rod-shaped nanoparticles, which can efficiently penetrate the ischemic brain for stroke treatment [20]. In this study, we further synthesized and characterized BA NPs for treatment of brain cancer. The anti-tumor activity of BA NPs on GBM cells in vitro and in vivo was studied. We found that BA NPs can effectively inhibit cell proliferation, block cell cycle in G0/G1 phase, and induce cell apoptosis. In addition, BA NPs slowed GBM growth in xenograft GBM mouse models and orthotopic tumor models. Collectively, our findings suggest that BA NPs may be a promising drug to inhibit the progress of GBM.

## Result and discussion

### Synthesis and characterization of BA NPs

**Fig. 1:**
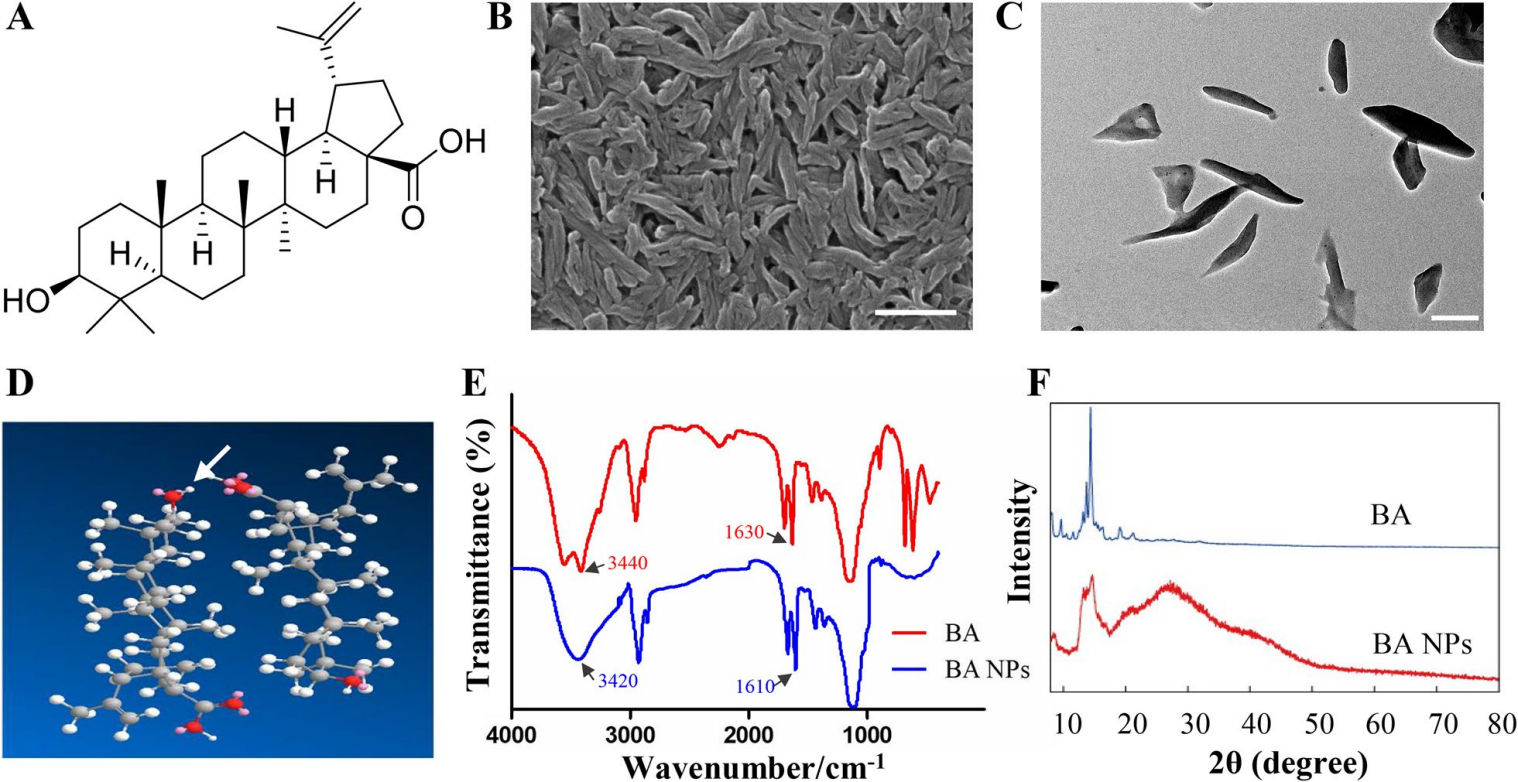
Characterization of BA NPs and BA NP formation. **A** Molecular structure of BA. **B** A representative SEM image showing the size and morphology of BA NPs in lyophilized form. Scale bar: 500 nm. **C** A representative TEM image showing the size and morphology of BA NPs. Scale bar: 200 nm. **D** Computational simulation shows the formation of hydrogen bonds between molecules (arrow showed). **E** FTIR analysis shows blue-shift of O–H and the C=O group after BA assembles into NPs. **F** XRD analysis shows that BA exists in the amorphous state in BA NPs

Recently, natural triterpenoids have received increasing attention due to their excellent biological activities. Among various triterpenoids, BA, a pentacyclic triterpenoid, has attracted much attention because of its significant anti-cancer effect (Fig. 1A) [13]. However, due to its poor water solubility and weak ability to pass through the BBB, its application in CNS tumors is greatly restricted [17]. In this study, BA NPs were prepared by solvent evaporation to improve their water solubility and BBB permeability. Scanning electron microscopy (SEM) and transmission electron microscopy (TEM) analysis showed that the BA NPs were rod-shaped particles in diameter of 60 nm and length of 400 nm (Fig. 1B, C). Dynamic light scattering (DLS) analysis showed that the zeta potential was − 6.78 mv (Additional file 1: Fig. S1). Using high performance liquid chromatography (HPLC), we found that 85% of BA NPs remain in the form of nanoparticles after being stored at room temperature for seven days (Additional file 1: Fig. S2). Furthermore, nanoparticles were identified under SEM after three months of storage at room temperature and away from light (Additional file 1: Fig. S3). These findings show that BA NPs have good stability in aqueous solution. Using Matertial Studio software, we built a water tank containing four BA molecules and optimized its structure. We found that BA molecules were polymerized within 3 ps (Additional file 1: Fig. S4), and the hydroxyl group in the molecule formed a hydrogen bond with the carboxyl group of another molecule (Fig. 1D). This finding suggests that formation of BA NPs is driven by intermolecular hydrogen bonds. Moreover, by Fourier transform infrared spectroscopy (FTIR), we found that for free BA, the peaks at 3440 and 1630 cm^−1^ were attributed to the stretching vibration of O–H and the C=O group, respectively. After being assembled into BA NPs, the two peaks were shifted to 3420 and 1610 cm^−1^ (blue-shift), indicating the formation of intermolecular hydrogen bonds, which further confirmed our previous conjecture (Fig. 1E). We further confirmed the self-assembly of BA NPs by X-ray powder diffraction (XRD) analysis. As shown in Fig. 1F, the XRD pattern of free BA reveals sharp characteristic peaks at 2θ of 9.6°, 14.5°, 18.9° and 22.3°, suggesting a high degree of crystallinity. In comparison, those peaks were absence in the XRD pattern of BA NPs, suggesting that BA exists in the amorphous state in BA NPs.

### BA NPs suppress glioma cell proliferation and arrest the cell cycles

**Fig. 2:**
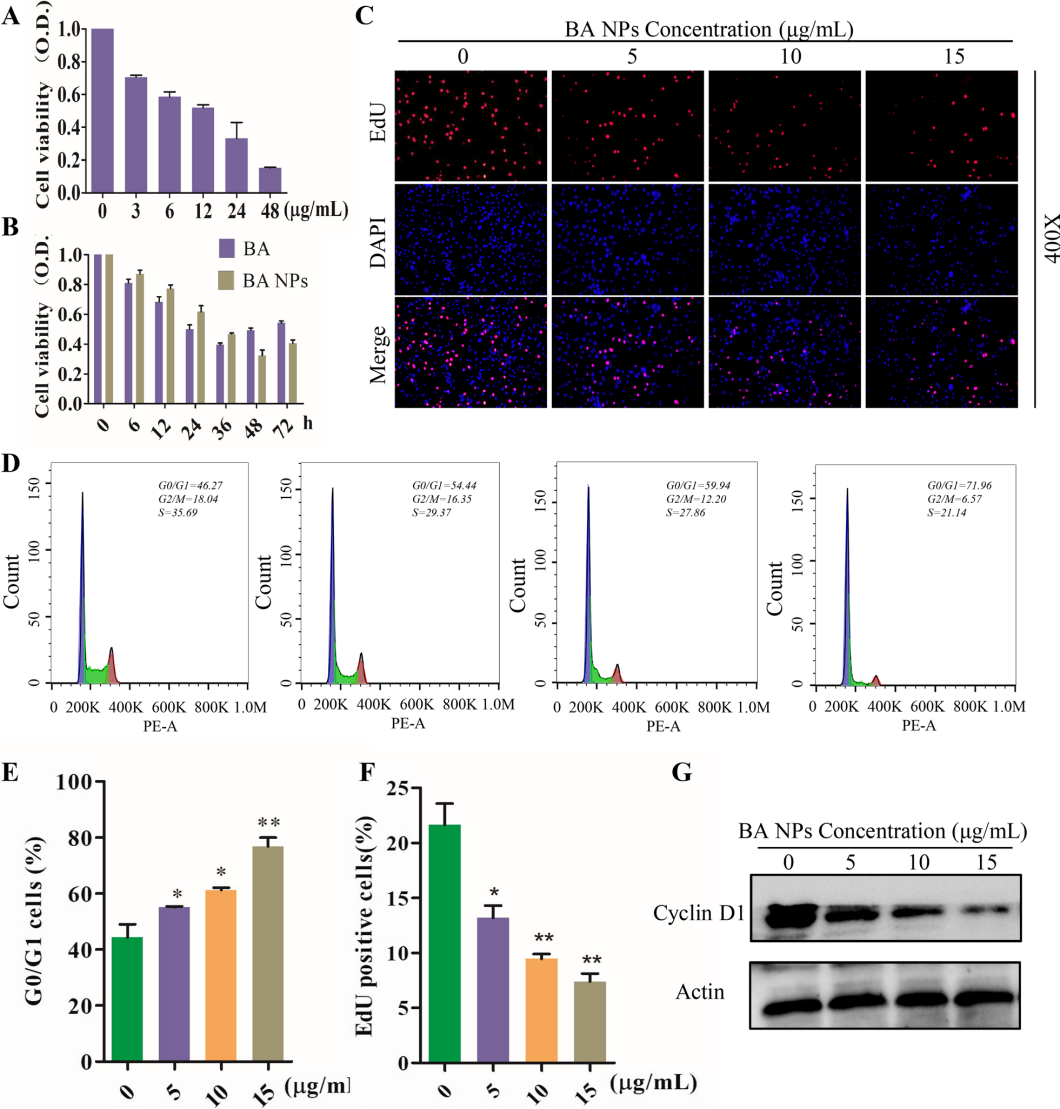
BA NPs suppresses the proliferation of human U87 cells in vitro. **A** Cell viability measured by CCK8 assay after BA treatment with various concentrations (0, 3, 6, 12, 24, 48 µg/mL). **B** Cell viability measured by CCK8 assay after BA treatment with various times (0, 6, 12, 24, 36, 48, 72 h). **C**, **F** EDU assay shown that BA NPs inhibited DNA synthesis in U87 cells. *P < 0.05, **P < 0.01. **D**, **E** Cell cycle distribution analysis showing the G0/G1 arrest by flow cytometry of U87 cells after been treated with various concentrations (0, 5, 10, 15 µg/mL) BA NPs. *P < 0.05, **P < 0.01. **G** Western blot analysis of the expression of Cyclin D1

To study the anti-proliferative activity of BA and BA NPs on GBM cells, CCK-8 was used. The results showed that BA has a dose-dependent inhibitory effect on the proliferation of U87 and A172 cells. The IC_50_ of BA in U87 and A172 cells was 12.1 µg/mL and 8.5 µg/mL respectively (Fig. 2A; Additional file 1: Fig. S5A). The time-dependent curve showed that the anti-proliferative activity of BA NPs is not significantly different from that of BA, but lasts longer (Fig. 2B; Additional file 1: Fig. S5B). The anti-proliferative activity was further examined by the EdU-DNA assay. We found that BA NPs significantly reduce in the percentage of EdU-positive cells in U87 cells (Fig. 2C, F). We studied whether BA NPs could prevent the proliferation of glioma cells through cell cycle arrest by PI staining following with flow cytometry analysis. Cell debris and late apoptotic cells was excluded through FSC and SSC gates. The results showed that BA NPs treatment resulted in the accumulation of cell cycle in G0/G1 phase (Fig. 2D, E). We further examined Cyclin D1, a key protein of the cell cycle signaling, by Western Blot and found its expression decreased at a dose-dependent manner (Fig. 2G; Additional file 1: Fig. S5C). Previous studies showed that BA has no obvious toxicity to normal tissues [21, 22]. To test if BA has potential toxicity to brain tissue, we determined the toxicity of BA to normal neuronal HT22 cells. We found that at 12 µg/mL, IC_50_ for U87 cells, BA did not show a detectable inhibitory effect on HT22 cell proliferation and the IC_50_ for HT22 cells is over 10 times greater than that for U87 cells (Additional file 1: Fig. S6). Collectively, those results suggest that BA NPs, while having limited toxicity to normal brain cells, efficiently inhibit the proliferation of glioma cells through cell cycle arrest.

### BA NPs induce glioma cell apoptosis

**Fig. 3:**
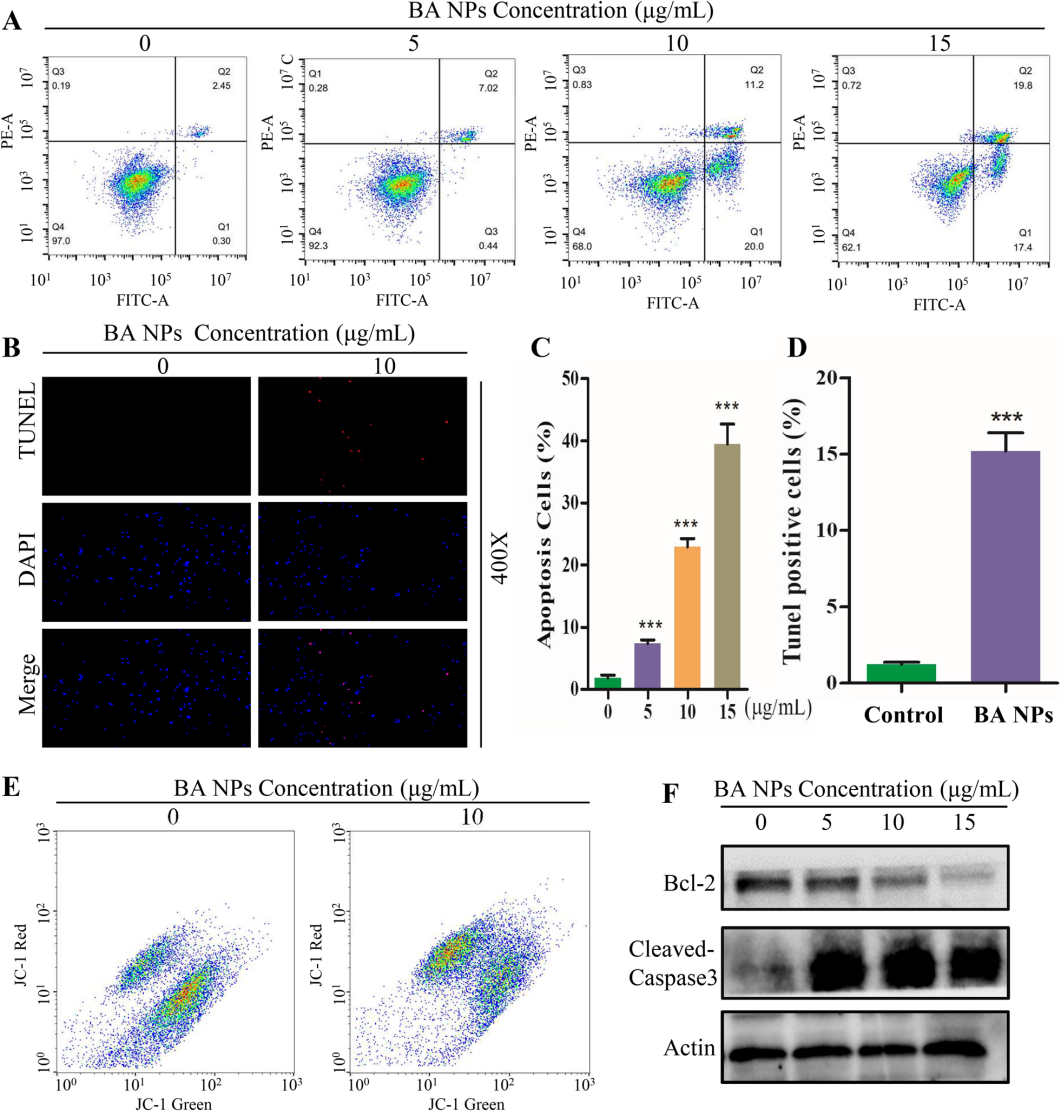
BA NPs induces cell apoptosis in U87 Cells. **A**, **C** Apoptosis Assay analysis of U87 cells by flow cytometry of U87 cells after treatment with various concentrations (0, 5, 10, 15 µg/mL) of BA NPs. ***P < 0.001. **B**, **D** Representative images of TUNEL staining after treatment with various concentrations (0, 10 µg/mL) of BA NPs. ***P < 0.001. **E** Mitochondrial membrane potential (ΔΨm) assay of U87 cells treated with BA NPs (Concentrations of BA NPs: 0, 10 µg/mL). **F** Cell apoptosis-related protein expression quantified with western blot

Through flow cytometry, we further explored the effect of BA NPs on U87 cell apoptosis. The results showed that 24 h after BA NPs treatment, the apoptotic ratio of U87 and A172 cells increased in a dose-dependent manner (Fig. 3A, C; Additional file 1: Fig. S7A, B). This finding was further confirmed by TUNEL staining analysis, which found that the proportion of TUNEL positive cells significantly increased after BA NPs treatment (Fig. 3B, D). One of the hallmark events in the early stage of apoptosis is the loss of the mitochondrial membrane potential (ΔΨm). Through JC-1 staining, we found that treatment with BA NPs induced significant loss of ΔΨm in U87 cells (Fig. 3E). Further Western Blot analysis showed that the expression levels of Bcl-2 decreased and Cleaved-caspase3 increased significantly (Fig. 3F; Additional file 1: Fig. S7C). Taken together, these results suggested that BA NPs could induce cellular apoptosis in U87 and A172 cells.

### BA NPs inhibit Akt/NFκB-p65 signaling through CB1 and CB2

**Fig. 4:**
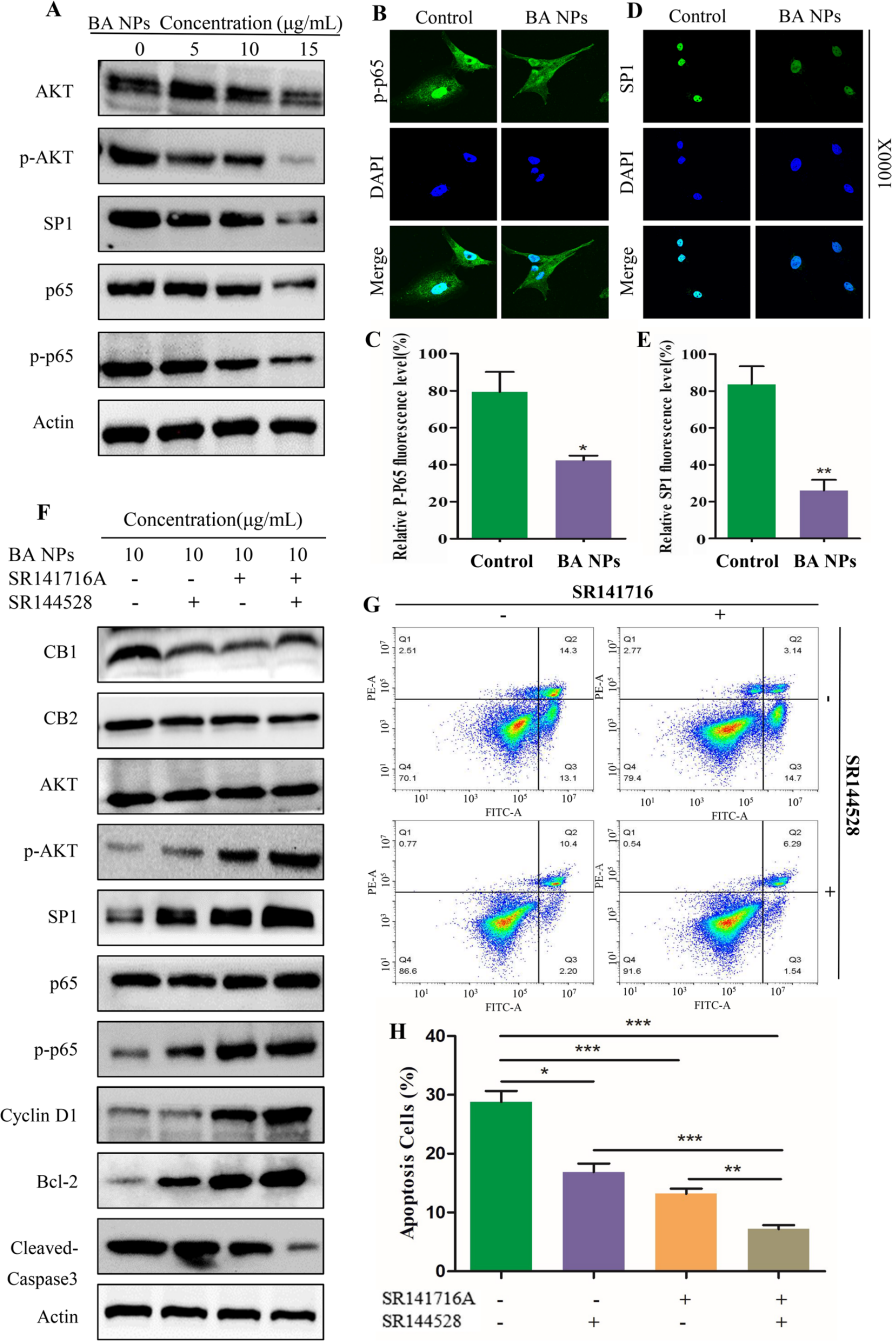
BA NPs suppress NFκB-p65 and SP1 signaling through CB1, CB2/Akt pathways. **A** Western blot assay of main protein expression of signaling pathways after BA NPs treatment in different concentration (0, 5, 10, 15 µg/mL). **B**, **C** Representative fluorescent images of p-p65 in U87 cells treated with BA NPs by confocal microscope. **D**, **E** Representative fluorescent images of SP1 in U87 cells treated with BA NPs treatment by confocal microscope. **F** Western blot analysis of the expression of the indicated proteins in U87 cells after treatment with BA NPs, CB1 INH (SR141716A) or CB2 INH (SR144528). G, H. Flow cytometry analysis (**G**) and quantification (**H**) of cell apoptosis after treatment with SR141716A or SR144528 prior to BA NPs treatment in U87 cells. ***P < 0.001; **P < 0.01; *P < 0.05

NFκB p65 and SP1 are considered to be closely related to the biological activity of BA [23, 24]. To study the mechanism accounting for the observed anti-glioma effects BA NPs, we assessed the changes of NFκB p65 and SP1 signaling in U87 and A172 cells after BA NP treatment using immunoblotting and immunofluorescence. The results showed that the expression of p-Akt, p-p65 and SP1 decreased in a dose-dependent manner (Fig. 4 A; Additional file 1: Fig. S8A). Immunofluorescence analysis showed that the fluorescence levels of p-p65 and SP1 in U87 cells treated with BA NPs were significantly lower than those in the control group, and the p-p65 nuclear cytotoxic translocation were inhibited by BA NPs (Fig. 4B–E). These results suggest that BA NPs induce cellular apoptosis through inhibition of the Akt/NFκB p65/SP1 signaling pathway.

CB1 and CB2 are two receptors of cannabinoid, which were found to be involved in the cannabinoid induced CNS effects. In our previous work, we found that CB1/CB2 is closely related to the role of BA in ischemic stroke [20]. SR141716A and SR144528 are reverse agonists of CB1 and CB2, respectively. As competitive inhibitors, SR141716A and SR144528 bind receptors at the protein level and do not reduce the expression of CB1 and CB2 (Fig. 4F). On the other hand, as the downstream signal molecules of CB1/CB2, the expression levels of p-STAT3 and SP1 changed, which confirmed that the two reverse agonists were effective (Additional file 1: Fig. S8B) [25, 26]. Then we explored the role of CB1/CB2 in the anti-glioma effects of BA NPs by cotreatment with BA NPs and SR141716A or SR144528 in U87 and A172 cells. We found that both the two reverse agonists could attenuate the effects of betulinic acid-induced down-regulation of Akt/NFκB-p65 pathway and downstream protein Cyclin D1 and Bcl-2 (Fig. 4F; Additional file 1: Fig. S8C). And the attenuation effect was maximized when both antagonists were used. Similar results were observed by detecting apoptosis by flow cytometry (Fig. 4G, H). These results indicated that CB1 and CB2 mediated BA NPs–induced effects on Akt/NFκB-p65 pathway in U87 and A172 cells.

### Cellular uptake, pharmacokinetics, and biodistribution of BA NPs

**Fig. 5:**
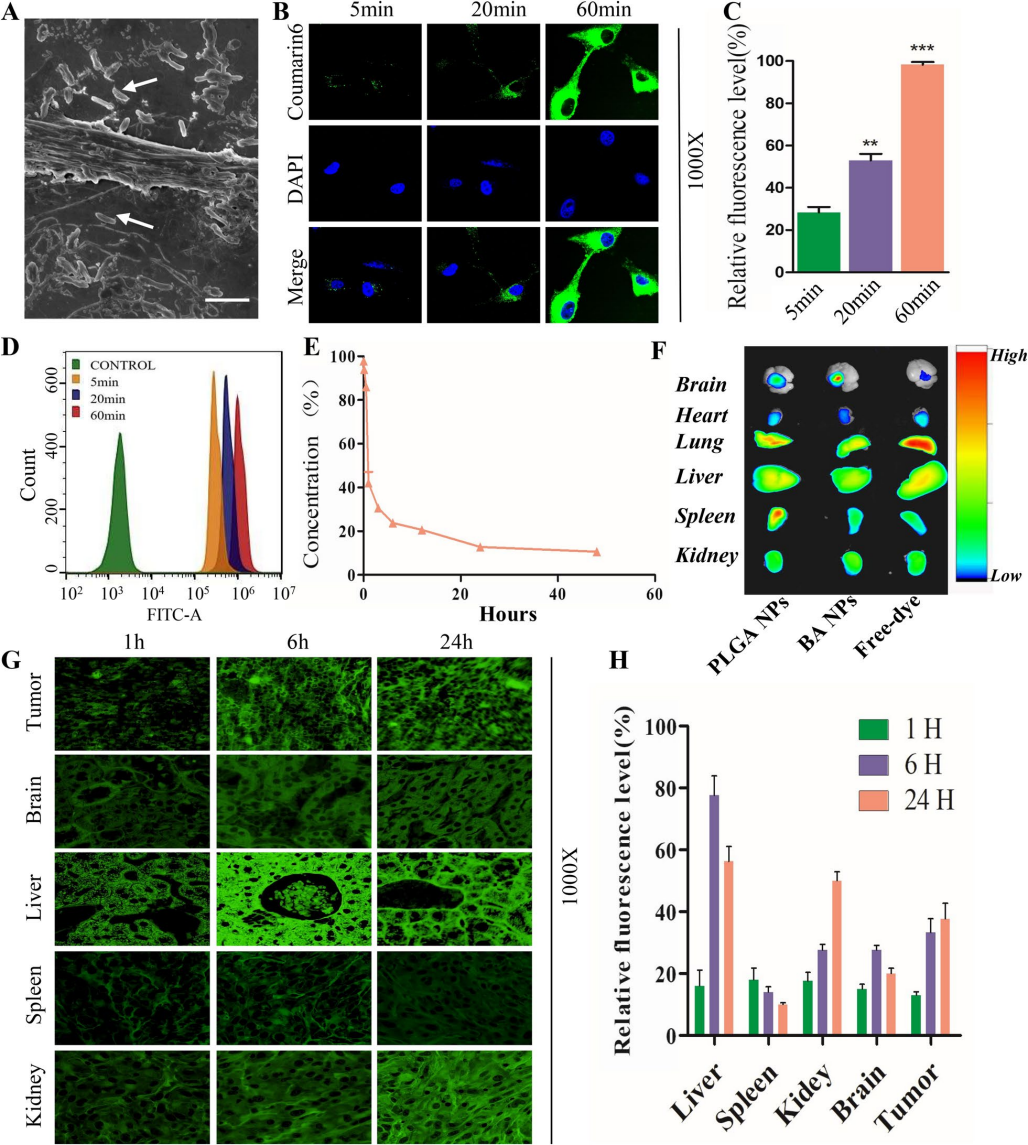
Cellular uptake and biodistribution of BA NPs in vitro and in vivo. **A** SEM pictures of U87 cells incubating with BA NPs for 4 h. Scale bar: 1000 nm. **B**, **C** Confocal microscopic pictures of U87 cells incubating with BA-coumarin-6-NPs (BA-C6 NPs) in different times (5, 20, 60 min). ***P < 0.001; **P < 0.01. **D** Flow cytometry analysis of U87 cells incubating with BA-coumarin-6-NPs in different times (0, 5, 20, 60 min). **E** The plasma concentration (C6 fluorescence intensity) changed with time after BA NPs injected from caudal vein. **F** IVIS image of the brain, heart, lung, liver, spleen, and kidney 8 h after IR780 labeled PLGA NPs, BA NPs and free dye injected into mice via tail vein. **G**, **H** Confocal microscope images of the tissues harvested from BA-C6 NPs injected mice euthanized at the indicated time points

We explored the uptake behavior of BA NPs by incubating BA NPs with U87 cells for 4 h, following with fixation with paraformaldehyde. By SEM, we found that BA NPs were able to efficiently attached to the surface of U87 cells and some of them were inserted into the cell membrane through their long axis, leading to membrane distortion and penetration (Fig. 5A). To further characterize cellular uptake of BA NPs, we synthesized BA NPs with encapsulation of coumarin-6 (C6), and the resulting C6-loaded BA NPs, or BA-coumarin-6-NPs (BA-C6 NPs) (Additional file 1: Fig. S9), were incubated with U87 cells. The uptake behavior of cells was monitored by fluorescence microscope and flow cytometry. We found that, at the 60 min, BA-C6 NPs spread the entire cytoplasm around the nucleus but not the nucleus (Fig. 5B, C). These results suggest that BA-C6 NPs were successfully internalized into tumor cells in a time-dependent manner. This finding was further confirmed by flow cytometry analysis (Fig. 5D).

We characterized the pharmacokinetics and biodistribution of BA NPs. To determine the pharmacokinetics, BA-C6 NPs were synthesized and administered into intracranial xenograft GBM mice via tail vein injection. The blood was collected at various time points and the blood concentration of BA-C6 NPs was detected based on C6 fluorescence. Figure 5E showed that the fluorescence intensity rapidly decreases within three hours after BA NPs injected from tail vein. To determine the biodistribution, BA NPs were synthesized with IR780, an infrared dye allowing for non-invasive imaging. Nude mice were inoculated with U87 cells. 4 weeks later, IR780-loaded BA NPs, or BA-IR780 NPs, were intravenously administered into the mice. Eight hours later, the mice were imaged using an IVIS system (Additional file 1: Fig. S10). We found that the signal in tumors increased significantly, which indicated that BA NP could reach the brain tumor tissue through BBB compared with PLGA-IR780 NPs and free IR780 (Fig. 5F; Additional file 1: Fig. S11). Lastly, to determine the distribution of BA NPs at various time points, BA-C6 NPs and free C6 were injected into tumor bearing mice through the tail vein. The mice were sacrificed at different time points. Tumors and major organs were collected for in vitro fluorescence imaging (Fig. 5G, H). We found that there was weak fluorescence signal in peripheral organs and limited signal in the brain and brain tumors (Additional file 1: Fig. S12). However, in mice injected with BA-C6 NPs, the fluorescence in the liver peaked at eight hours after NP administration, while the fluorescence in the kidney increased with time, and the fluorescence signal in brain tumors also increased with time. To eliminate the possibility that the observed signal was from free dye release from NPs, we determined the release profile of C6-loaded BA NPs and found that the release of C6, which is highly hydrophobic, from BA NPs is limited (Additional file 1: Fig. S13). These results suggesting that the accumulation of BA NPs continuously increased in tumor tissues with time (Fig. 5G, H).

### BA NPs inhibited tumor growth in glioma xenograft models

**Fig. 6:**
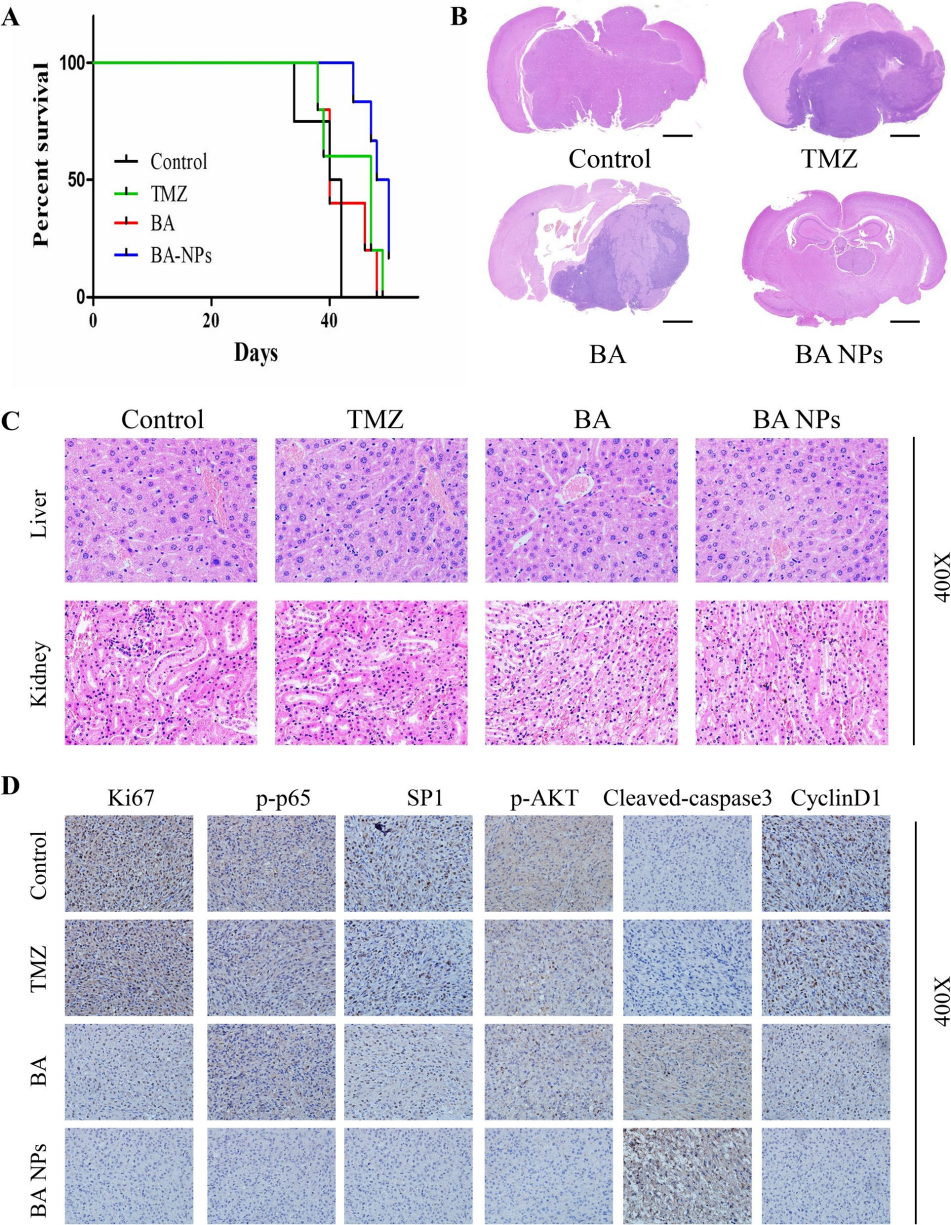
BA NPs Suppressed Tumor Growth in intracranial Xenograft model. **A** Mice survival is shown by Kaplan-Meier curves. **B** Representative images of mouse brain sections from mice that were treated with PBS, TMZ, free BA or BA NPs. Scale bar: 1.0 mm. **C** Pathological section examination stained with H&E of liver and kidney under a microscope. **D** IHC analyses of Ki67, p-p65, SP1, p-Akt, Cleaved-caspase 3 and Cyclin D1 in mice tumor sections

We assessed BA NPs for glioma treatment in vivo. Nude mice were intracranially inoculated with U87 cells. 2 weeks later, the mice were randomly grouped and received treatment of PBS, TMZ, free BA, or BA NPs. BA NPs and free BA were given at a dose of 20 mg/kg for 3 times a week. After 3 weeks of treatment, 3 mice in each group were randomly selected and euthanized, the brain and major organs were taken for hematoxylin–eosin (H&E) staining and immunohistochemical analysis. The remaining mice were monitored for survival. We found that compared with that for the group receiving PBS, the survival of mice receiving free BA and TMZ group was significantly increased, while formulation BA into NPs further enhanced the survival benefit (Fig. 6A). Consistently, H&E staining showed that the sizes of tumor in the BA NP-treatment group were much smaller than those in the other two groups (Fig. 6B). At the meanwhile, H&E analysis of major organs including liver, kidney, heart and spleen did not identify detectable toxicity (Fig. 6C; Additional file 1: Fig. S14), suggesting that intravenous administration of BA NPs has limited systemic toxicity.

Finally, the brain tumors dissected from mice in each group were analyzed by immunohistochemistry to further evaluate the anti-tumor effect. We found that the amount of proliferative tumor cells in the BA NP-treatment group, which were identified by Ki67 staining, was much lower than those in in PBS, TMZ and free BA groups (Fig. 6D). Furthermore, consistently with our in vitro finding (Fig. 4A), we found that, compared with the other two groups, the expressions levels of p-Akt, SP1 and p-p65 in tumors in BA NP-treatment group were significantly lower (Fig. 6D). These findings suggest that BA NPs after intravenous administration effectively inhibited glioma development through inhibition of the Akt/NFκB-p65 signaling.

## Conclusions

In summary, we demonstrated that BA NPs have strong anti-glioma effects and could effectively cross the BBB in an intracranial xenograft model. The therapeutic effects of BA NPs are mainly mediated by CB1/CB2 through suppression of the Akt/NFκB-p65 signaling. Due to their significant anti-glioma efficacy and excellent safety, BA NPs have the potential to be translated into clinical applications to improve clinical management of GBM.

### Methods

1. *Cell culture* Human glioblastoma cell lines (U87 and A172) were acquired from the Cell Bank Culture Library of the Chinese Academy of Sciences (Shanghai, China). Cells were cultured at 37 °C and 5% CO_2_ in high-glucose DMEM (Gibco, USA) along with 10% fetal bovine serum (FBS) and 1% penicillin/streptomycin.
2. *Drugs and antibodies* Betulinic acid was purchased from Nanjing Spring and Autumn Biological Products Co., Ltd [purity (98%), China]. The antibodies included the following: anti-Phospho-p65 (Ser536) [#93H1, Cell Signaling Technology], anti- p65 [#8242S, Cell Signaling Technology], anti-Phospho-Akt [#4060, Cell Signaling Technology], anti-Akt [#4691, Cell Signaling Technology], anti-Phospho-Stat3 (Tyr705) [#9145S, Cell Signaling Technology], anti-SP1 [GTX110593, Genetex], anti-CB1 [GTX110219, Genetex], anti-CB2 [GTX23561, Genetex], anti-Bcl-2 [127,891-AP, Proteintech], anti-Cyclin D1[60186-1-Ig, Proteintech], anti-Cleaved-caspase3 [19677-1-AP, Proteintech].
3. *Synthesis of BA NPs* 10 mg BA was dissolved in a mixed organic solution of ethyl acetate (1 mL) and dimethyl suolfoxide (DMSO) (0.02 mL), and added dropwise to 3 mL of 2.5% polyvinyl alcohol (PVA) solution. The obtained emulsion was sonicated on ice for 60 s (power: 120 w, 10 s on, 10 s off), poured into 35 mL of 0.3% PVA aqueous solution, and stirred overnight at 300 rpm. BA NPs were collected by centrifugation at 18,000 rpm for 30 min. Then, the supernatant was discarded, and the particles were suspended in 40 mL of water and collected by centrifugation at 18,000 rpm for 30 min to obtain NPs. Finally, the particles were resuspended in 1 mL of water, and stored at 4 °C. For the synthesis of BA NPs with hydrophobic cargo (including coumarin-6 and IR780), the selected cargo can be dissolved in ethyl acetate mixture with BA in the initial step.
4. *Scanning electron microscopy (SEM)* Gold plating was carried out on the samples under vacuum and argon atmosphere with sputtering current of 20 mA for 100 s. (auto fine coater JFC 1600, JEOL Ltd., Japan). SEM imaging was performed with Zeiss SIGMA field emission scanning electron microscope (Zeiss SIGMA,Carl Zeiss AG, UK) with 5 kV acceleration voltage and 10,000 times magnification.
5. *Transmission electron microscopy (TEM)* NPs resuspended in 10 µL water were applied to holey carbon-coated copper grids (SPI, West Chester, PA, USA). A filter paper was used to absorb the NPs after 5 min. The grids were left at fume hood until completely dried and then visualized by using a JEOL 1230 transmission electron microscope (JEOL Ltd., Japan) at 100 kV.
6. *Dynamic light scattering (DLS)* BA NPs was diluted to 1 mg/mL aqueous solution. The hydration diameter and zeta potential were measured by Dynamic Light Scatterer (Zetasizer Nano ZSP, Malvern instruments Ltd., UK).
7. *Fluorescent imaging* IR780 is a fluorescent dye employed to in vivo imaging. IR780-loaded BA NPs were administered intravenously through the tail vein 4 week after balb/c nude mice intracranially inoculated with U87 cells. Mice were imaged by Bruker Xtreme BI imaging system with excitation wavelength of 730 nm and emission wavelength of 830 nm for free IR780 or IR780-loaded NPs 6 h after injection. Mice were sacrificed to isolate the brain and other organs and imaged by IVIS. Fluorescence intensity in each brain was quantified using Living Image 3.0 (Xenogen).
8. *Drug stability test* BA NPs were placed in dark at room temperature, and 10 µL of BA NPs were taken at 1, 3 and 7 d. They were dissolved in methanol, and the concentration of BA was detected by HPLC.
9. *Cell viability assay* Cell Counting Kit-8 (CCK-8) was used to determine the anti-proliferative activity of BA and BA NPs according to the protocol of Target Mol Inc. Inoculate U87 or A172 cells in a 96-well plate (10 000 cells/well), then treated with different concentrations of drugs to incubate for 24 h. 10 µL CCK8 was incubated to each well at 37 °C for 1 h. And the OD value at 450 nm was tested with a microplate reader.
10. *EdU-DNA synthesis assay* Cell-Light-EdU-Apollo567 in vitro culture kit (RiboBio, Guangzhou, China) was used to detected the cell growth. 10,000 cells of U87 were seeded in a 96-well plate containing 100 µL DMEM and treated with 0, 5, 10, 15 µg/mL BA NPs for 24 h. The cells were cultured in 50 µL EdU medium for 2 h and fixed with 4% paraformaldehyde for 30 min. Subsequently, 100 µL 1X Apollo^®^ reaction cocktail was added, incubated for 30 min, and then counterstained with 1 × DAPI for 30 min in the dark. A fluorescence microscope (Olympus BX51, Japan) was used to observe the fluorescence images of DAPI and EdU.
11. *Cell cycle assay* After treating the cells with different concentrations of BA NP for 24 h, the cells were harvested with 0.25% trypsin. Next, the cells were fixed in 70% cold ethanol at − 20 °C for 12 h. Then the fixed cells were washed three times with PBS and incubated with PBS containing RNase for 30 min. Finally, the cells were stained with propidium iodide (PI) for 15 min in the dark, and the cell cycle was measured with a flow cytometer (Beckman, USA), and quantitative analysis was performed with FlowJo v10 software.
12. *Mitochondrial membrane potential (ΔΨm) assay* The cells were incubated with BA or BA NPs. The Δψm variation was obtained by using a flow cytometer to capture the fluorescence intensity of the cells after JC-1 staining (GUYOO, Shanghai, China).
13. *Apoptosis assay* After treating the cells with different concentrations of BA NPs for 24 h, the cells were harvested with 0.25% trypsin. Then, 800ul of FITC-containing binding solution was added to the cell pellet, and incubated for 30 min in the dark. Finally, the cells were stained with propidium iodide (PI) for 15 min in the dark and the cell cycle was measured with a flow cytometer (Beckman, USA). Quantitative analysis was performed with FlowJo v10 software.
14. *Western Blot Analysis* U87 or A172 cells were lysed in a modified RIPA buffer (NO. P0013B, Beyotime Biotechnology, China) on ice for about 30 min, then centrifuged at 12,000 rpm for 15 min. The concentration of the sample was quantitatively determined by BCA protein assay. The lysate was mixed with loading buffer after heated at 100 °C for 5 min. In brief, equal protein amount was loaded on 8–15% SDS-PAGE and then transferred to a nitrocellulose membrane. Next PVDF membrane was blocked in 5% non-fat milk for 1 h and incubated with primary antibody at 4 °C overnight and secondary antibodies for 1 h.
15. *Immunofluorescence staining* Cells were fixed with 4% paraformaldehyde for 30 min, permeated with 0.2% tritonx-100 (PBS) for 15 min at room temperature, and the slides were washed 3 times with PBS. 1% BSA was added dropwise to the glass slide at room temperature for 30 min, then sufficient diluted primary antibody was added, placed it in a humid box, and incubated overnight at 4 °C. A secondary antibody (Antgene, Wuhan, China) was incubated for 1 h in a humid chamber at 37 °C in the dark. The slides were mounted with DAPI anti-fluorescence quencher. Finally, the glass slide was imaged under a fluorescence microscope (Japanese olympusbx51).
16. *Intracranial Xenograft model* U87 cells were suspended in PBS at a concentration of 1 × 105 cells/µL and stereotactically implanted into the right ventricle of 6-week-old Balb/c nude mice. Two weeks after implantation, the mice were divided into Control, TMZ, BA and BA NP group randomly. PBS, TMZ, BA, or BA NPs were injected into the tail vein, three times a week for 3 consecutive weeks. In order to analyze the survival rate, the mice were regularly monitored, and when severe neurological symptoms and/or significant weight loss (more than 20% of their body weight) appeared, they were sacrificed. The whole mouse brain was obtained and weighed. All samples were fixed with 4% polyoxymethylene. The brain was saved for further analysis and embedded in paraffin. The Animal Protection and Utilization Committee of Wuhan University People’s Hospital approved the above-mentioned animal experiment.
17. *Statistical analysis* All experiments were carried out at least 3 times. SPSS version 24 was used for data analysis. Significance between groups was assessed by Student’s t test. The data are expressed as mean ± standard deviation (SD). *P < 0.05 indicates that the difference is statistically significant.

## Supplementary Information

**Additional file 1**: **FigureS1**. Zeta potential of BA NPs. **Figure S2**. Relativeconcentration of betulinic acid in dark at room temperature. **Figure S3**. Representative SEMimage of BA NPs stored in normal temperature and dark house for 3 months. Scalebar: 500 nm. **FigureS4**. BA combination mode diagram by Matertial Studio.**Figure S5**. BANPs suppresses the proliferation of human A172 cells in vitro. (A) Cellviability measured by CCK8 assay after BA treatment with variousconcentrations. (B) Cell viability measured by CCK8 assay after BA treatmentwith various times. (C) Cell cycle protein expression quantified with Westernblot. **Figure S6**. Cellviability of HT22 cells measured by CCK8 assay after BA treatment with variousconcentrations. **FigureS7**. BA NPs induces cell apoptosis in A172 Cells. (A,B) Apoptosis Assay analysis of A172 cells by flow cytometry of A172 cells aftertreatment with various concentrations of BA NPs. ***P < 0.001. (C) Cell apoptosis-relatedprotein expression quantified with western blot. **Figure S8**. BA NPs suppress NFκB-p65and SP1 signaling through CB1, CB2/Akt pathways. (A) Western blot assay of mainprotein expression of signaling pathways after BA NPs treatment. (B ) Theexpression level of p-STAT3 and SP1, which are downstream signaling of CB1 andCB2 pathways. (C) Western blot of main protein expression of signalling pathwaysafter BA, CB1 INH (SR141716A) and CB2 INH (SR144528) treatment. **Figure S9**. TheSEM image of BA-C6 NPs. Scale bar: 200nm. **Figure S10**. IVIS imaging ofBA-IR780 NPs injected intracranial xenograft GBM mouse. **Figure S11**.TheSEM image of PLGA-IR780 NPs. scare bar: 500 nm. **Figure S12**. Ex vivo fluorescenceimaging of the free dye tissues injected harvested in 8 hours. Scale bar: 20 μm.**Figure S13**. Therelease curve of BA-C6 NPs. **FigureS14**. HE stain of the heart and spleen.

## Data Availability

The data used to support the findings of this study are available from the corresponding author upon request.
