# Supplementary material for: Betulinic acid self-assembled nanoparticles for effective treatment of glioblastoma
Source: J Nanobiotechnology. 2022 Jan 21;20:39. doi: 10.1186/s12951-022-01238-7 (PMC8781388; doi:10.1186/s12951-022-01238-7)
Supplement: Supplementary file 1 — Additional file 1: FigureS1. Zeta potential of BA NPs. Figure S2. Relativeconcentration of betulinic acid in dark at room temperature. Figure S3. Representative SEMimage of BA NPs stored in normal temperature and dark house for 3 months. Scalebar: 500 nm. FigureS4. BA combination mode diagram by Matertial Studio.Figure S5. BANPs suppresses the proliferation of human A172 cells in vitro. (A) Cellviability measured by CCK8 assay after BA treatment with variousconcentrations. (B) Cell viability measured by CCK8 assay after BA treatmentwith various times. (C) Cell cycle protein expression quantified with Westernblot. Figure S6. Cellviability of HT22 cells measured by CCK8 assay after BA treatment with variousconcentrations. FigureS7. BA NPs induces cell apoptosis in A172 Cells. (A,B) Apoptosis Assay analysis of A172 cells by flow cytometry of A172 cells aftertreatment with various concentrations of BA NPs. ***P < 0.001. (C) Cell apoptosis-relatedprotein expression quantified with western blot. Figure S8. BA NPs suppress NFκB-p65and SP1 signaling through CB1, CB2/Akt pathways. (A) Western blot assay of mainprotein expression of signaling pathways after BA NPs treatment. (B ) Theexpression level of p-STAT3 and SP1, which are downstream signaling of CB1 andCB2 pathways. (C) Western blot of main protein expression of signalling pathwaysafter BA, CB1 INH (SR141716A) and CB2 INH (SR144528) treatment. Figure S9. TheSEM image of BA-C6 NPs. Scale bar: 200nm. Figure S10. IVIS imaging ofBA-IR780 NPs injected intracranial xenograft GBM mouse. Figure S11.TheSEM image of PLGA-IR780 NPs. scare bar: 500 nm. Figure S12. Ex vivo fluorescenceimaging of the free dye tissues injected harvested in 8 hours. Scale bar: 20 μm.Figure S13. Therelease curve of BA-C6 NPs. FigureS14. HE stain of the heart and spleen. [file 12951_2022_1238_MOESM1_ESM.pdf]

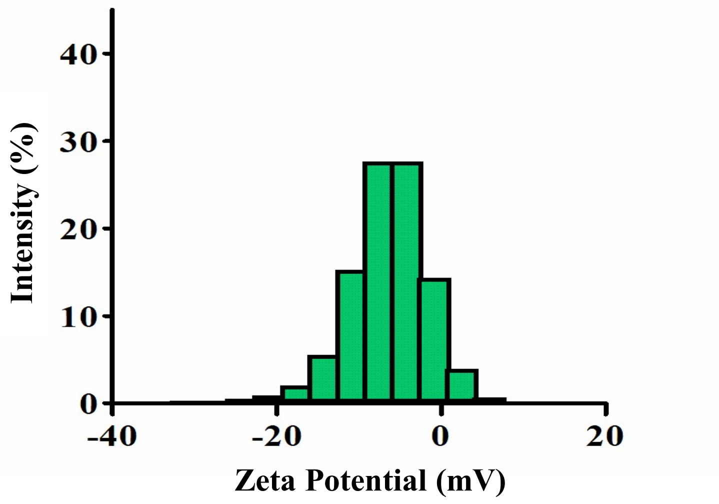

Figure S1. Zeta potential of BA NPs.

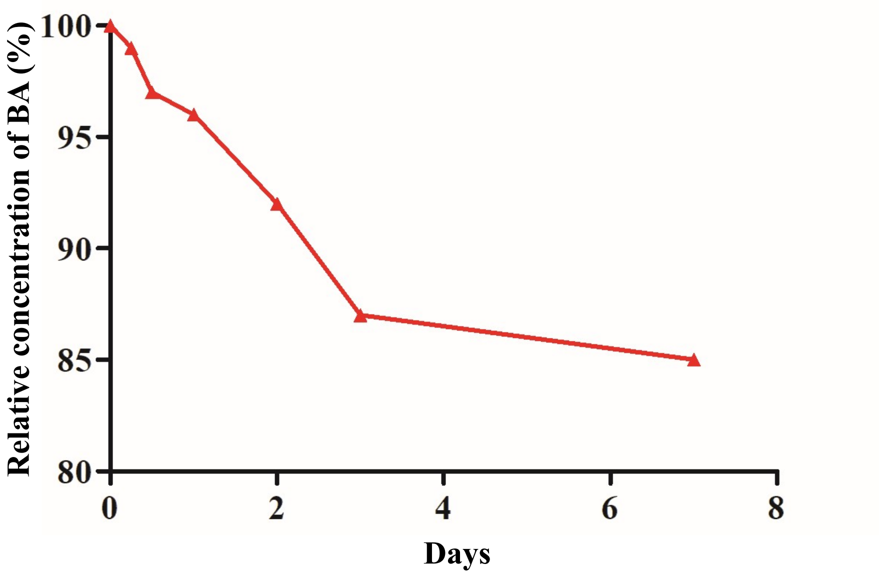

Figure S2. Relative concentration of betulinic acid in dark at room temperature.

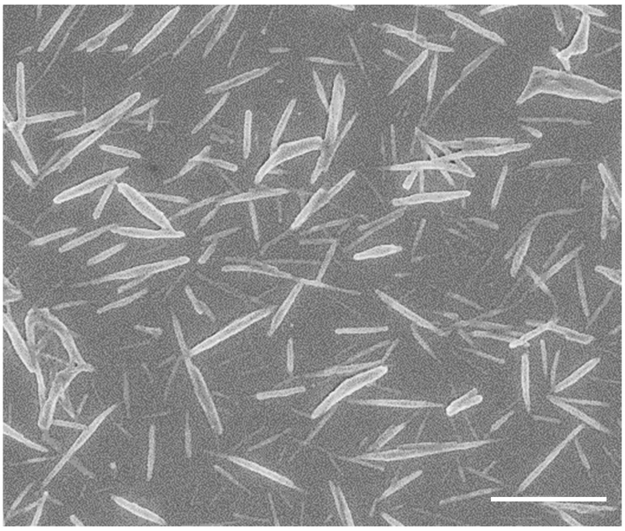

Figure S3. Representative SEM image of BA NPs stored in normal temperature and dark house for 3 months. Scale bar: 500 nm.

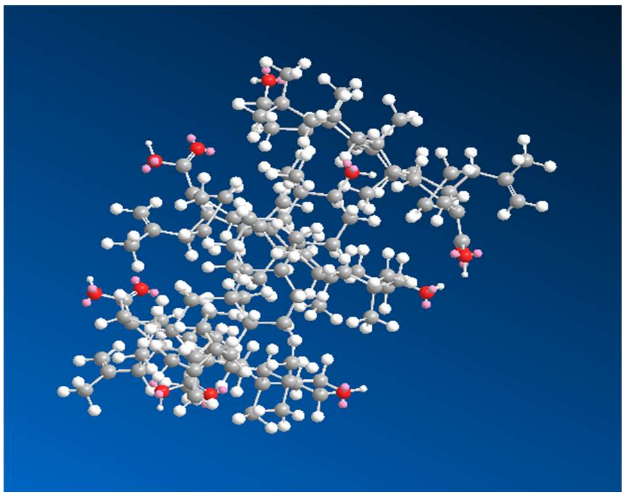

Figure S4. BA combination mode diagram by Matertial Studio.

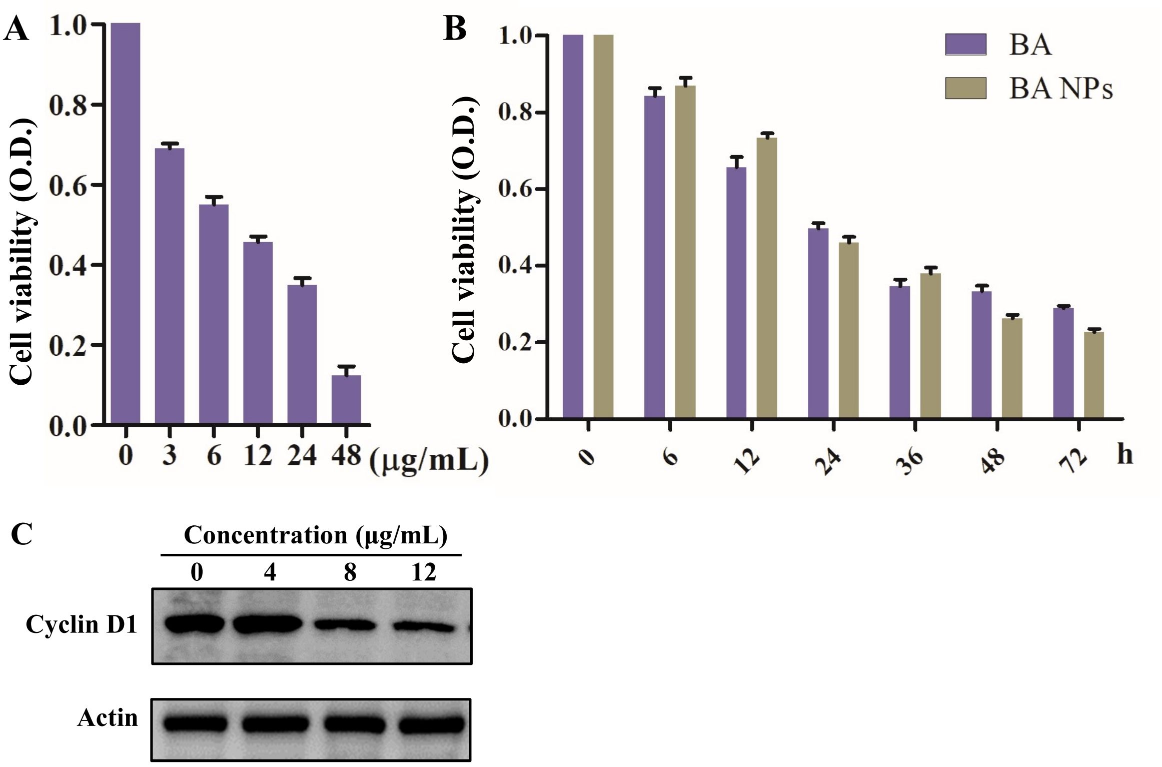

Figure S5. BA NPs suppresses the proliferation of human A172 cells in vitro. (A) Cell viability measured by CCK8 assay after BA treatment with various concentrations. (B) Cell viability measured by CCK8 assay after BA treatment with various times. (C) Cell cycle protein expression quantified with Western blot.

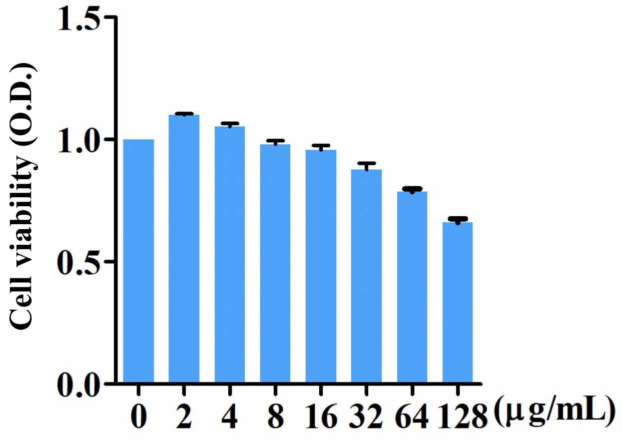

Figure S6. Cell viability of HT22 cells measured by CCK8 assay after BA treatment with various concentrations.

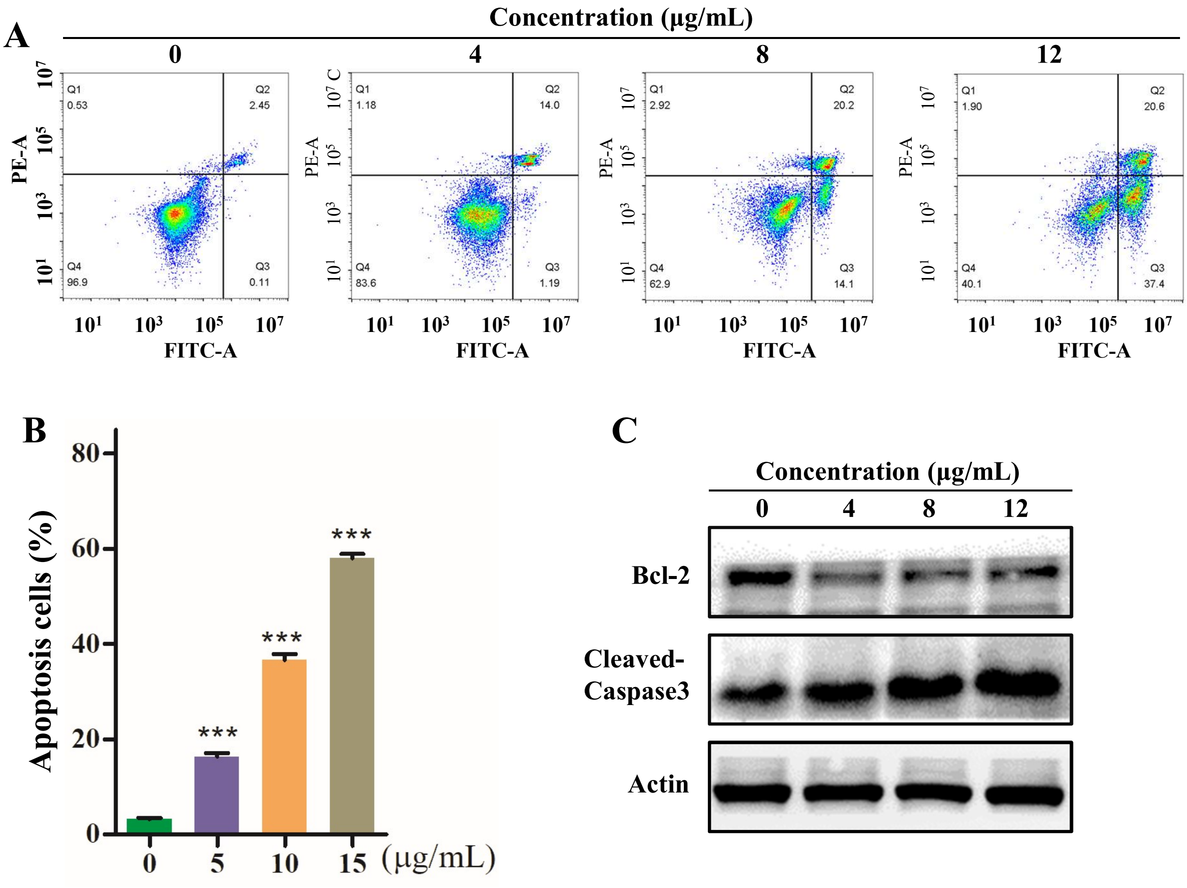

Figure S7. BA NPs induces cell apoptosis in A172 Cells. (A, B) Apoptosis Assay analysis of A172 cells by flow cytometry of A172 cells after treatment with various concentrations of BA NPs. *** $P < 0.001$. (C) Cell apoptosis-related protein expression quantified with western blot.

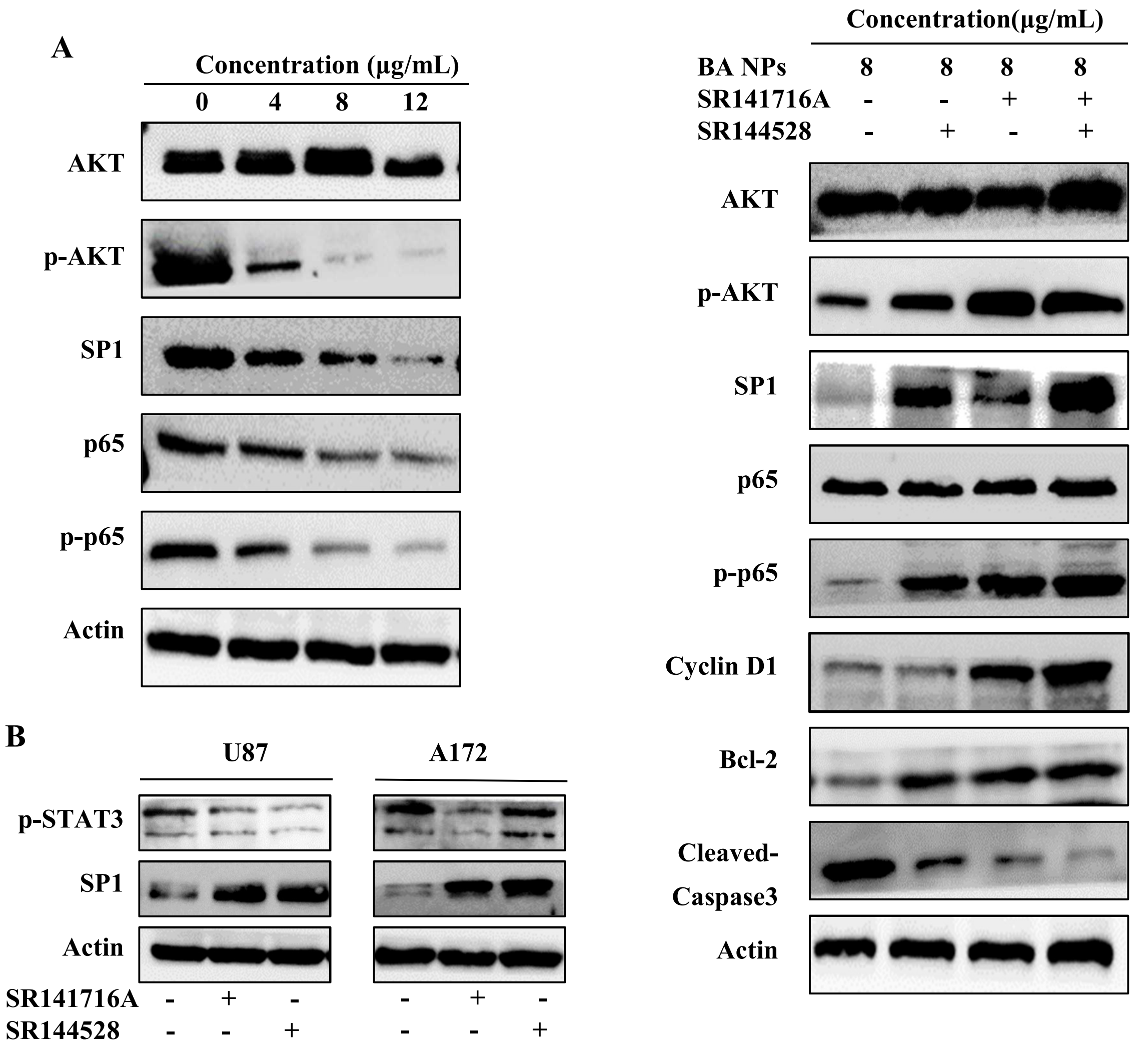

Figure S8. BA NPs suppress NFκB-p65 and SP1 signaling through CB1, CB2/Akt pathways. (A) Western blot assay of main protein expression of signaling pathways after BA NPs treatment. (B) The expression level of p-STAT3 and SP1, which are downstream signaling of CB1 and CB2 pathways. (C) Western blot of main protein expression of signaling pathways after BA, CB1 INH (SR141716A) and CB2 INH (SR144528) treatment.

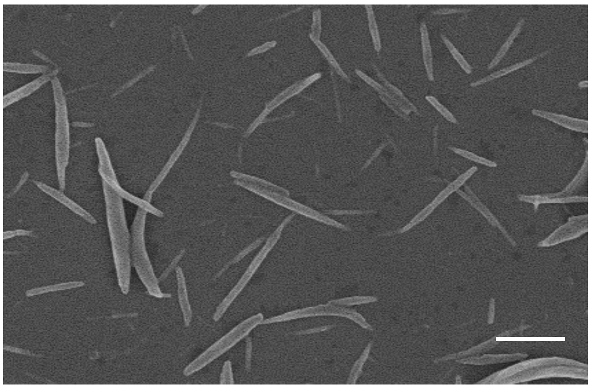

Figure S9. The SEM image of BA-C6 NPs. Scale bar:200nm.

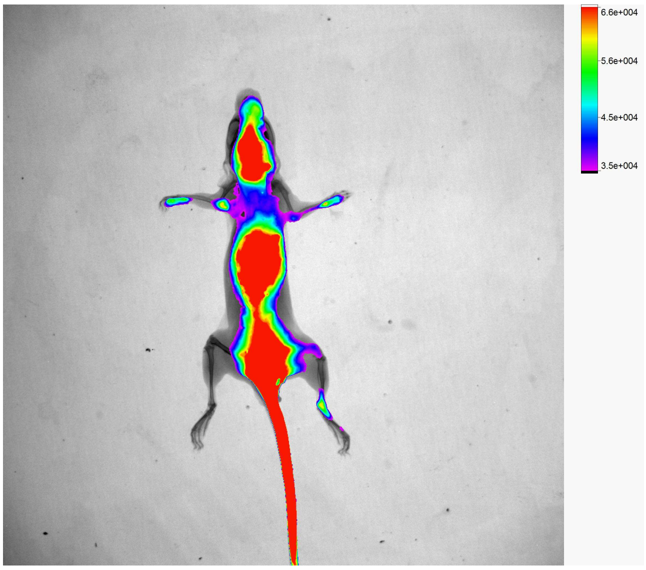

Figure S10. IVIS imaging of BA-IR780 NPs injected intracranial xenograft GBM mouse.

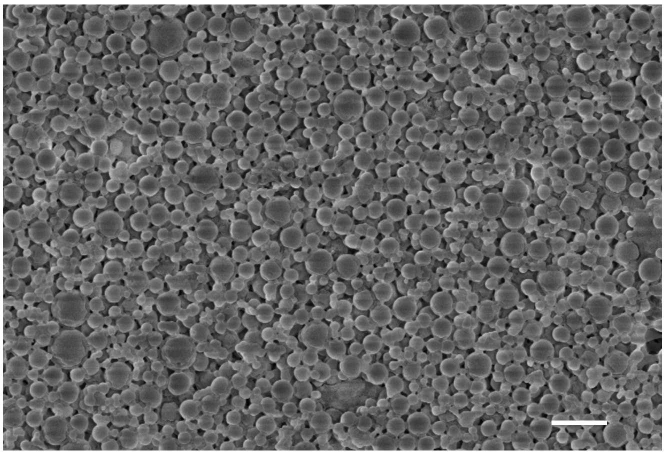

Figure S11. The SEM image of PLGA-IR780 NPs. scare bar:500nm.

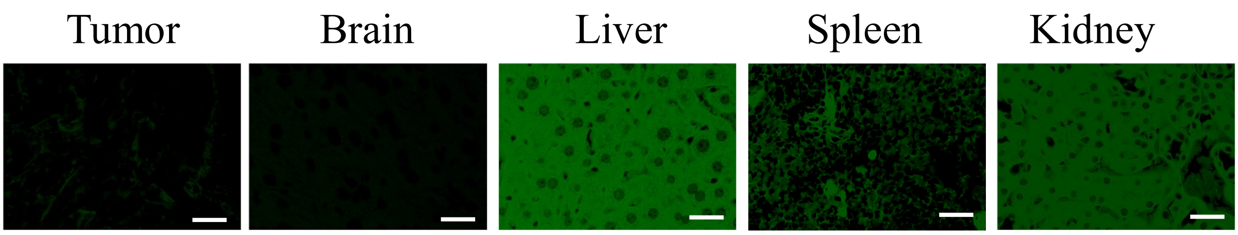

Figure S12. Ex vivo fluorescence imaging of the free dye tissues injected harvested in 8 hours. Scale bar: 20μm.

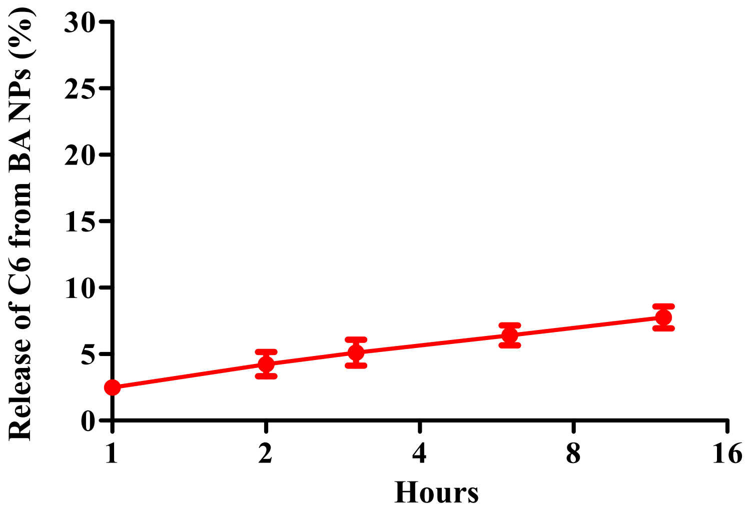

Figure S13. The release curve of BA-C6 NPs.

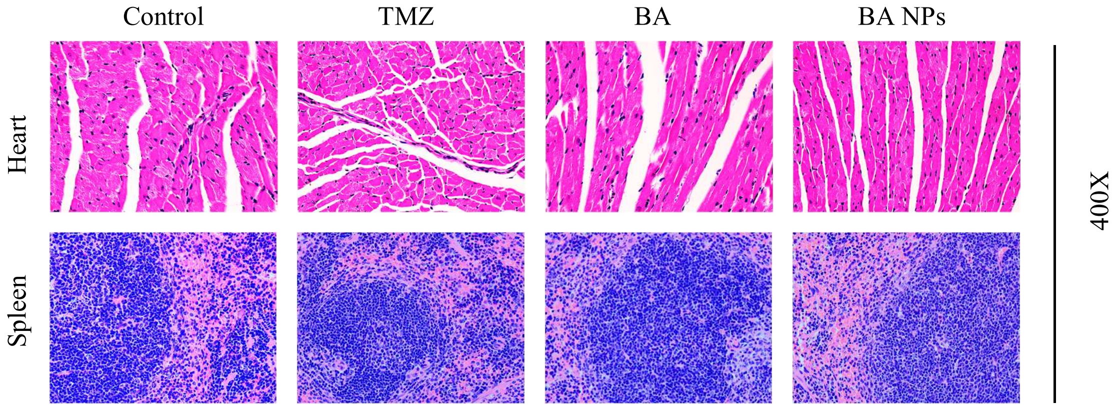

Figure S14. HE stain of the heart and spleen.
